# Diagnostic performance and inter-observer reproducibility of glomerular PLA2R immunohistochemistry in primary membranous nephropathy: a multi-laboratory blinded observer study

**DOI:** 10.1186/s12882-026-05233-0

**Published:** 2026-07-28

**Authors:** Birgitte G. Tougaard, Søren Krag, Eva Gravesen, Mie Emilie Kristensen, Kirsten Madsen, Lene Buhl Riis, Dorrit Krustrup, Per Ivarsen

**Affiliations:** 1 Department of Renal Medicine, Aarhus University Hospital, Palle Juul-Jensens Boulevard 99, Aarhus N, 8200 Denmark; 2 Department of Pathology, Aarhus University Hospital, Aarhus, Denmark; 3 Department of Pathology, Copenhagen University Hospital – Herlev and Gentofte, Herlev, Denmark; 4 Department of Pathology, Odense University Hospital, Odense, Denmark; 5 Department of Clinical Medicine, Aarhus University, Aarhus, Denmark

**Keywords:** Membranous nephropathy, Phospholipase A2 receptor, Immunohistochemistry, Diagnostic performance, Inter-observer agreement, ICC, ONEST

## Abstract

**Background:**

PLA2R immunohistochemistry (IHC) and serum anti-PLA2R antibodies are key diagnostic tools in PLA2R-associated membranous nephropathy (MN). However, diagnostic performance and inter-observer reproducibility of PLA2R IHC across different laboratory protocols and cut-offs remain insufficiently validated. We performed local validation of two routine PLA2R IHC protocols and report the methodological considerations that emerged, including the interaction between cut-off selection, diagnostic accuracy, and observer reproducibility.

**Methods:**

In a multi-laboratory study, kidney biopsies from patients with primary MN (pMN), secondary MN, and non-MN controls were stained using two routine PLA2R IHC protocols (Aarhus University Hospital [AUH] and Herlev Hospital [HEH]). Seven blinded observers scored granular capillary wall staining intensity on a 0–3 scale. Diagnostic performance was assessed at a prespecified cut-off (≥ 2) and at protocol-specific optimal cut-offs determined by Youden’s J index. Inter-observer agreement for the ordinal scale was quantified using intraclass correlation coefficients (ICC). Reproducibility of dichotomized classifications was evaluated using the ONEST framework, examining overall percent agreement (OPA) across increasing numbers of observers.

**Results:**

At the prespecified cut-off (≥ 2), sensitivity/specificity was 70%/70% for AUH and 55%/100% for HEH. Applying Youden’s J calculated optimal cut-offs (AUH: ≥3; HEH: ≥1) yielded sensitivity/specificity of 65%/100% for AUH and 65%/93% for HEH. Single-rating reliability was good-to-excellent overall [ICC (2,1) = 0.85], with protocol-specific differences (AUH = 0.78; HEH = 0.93), and the seven-observer panel demonstrated excellent reliability [ICC (2,7) = 0.96–0.99]. ONEST analyses demonstrated higher agreement for HEH at the prespecified cut-off (OPA 93% vs. 60%) and earlier stabilization of agreement curves, whereas AUH remained sensitive to panel size throughout the seven-observer range. Diagnostic accuracy and reproducibility were not fully aligned for the HEH protocol: Youden’s J favored cut-off ≥ 1, whereas reproducibility was highest at ≥ 2.

**Conclusion:**

PLA2R IHC provides clinically useful diagnostic information for PLA2R-associated MN, but performance and reproducibility are protocol-dependent and were optimized at different cut-offs. Laboratories should therefore validate both dimensions locally rather than adopting a single fixed cut-off. We propose a stepwise framework combining Youden’s J and ONEST as a practical guide for laboratories implementing PLA2R IHC.

**Supplementary Information:**

The online version contains supplementary material available at 10.1186/s12882-026-05233-0.

## Introduction

Membranous nephropathy (MN) is a leading cause in adult nephrotic syndrome [1]. Identification of phospholipase A2 receptor (PLA2R) as a major target antigen in primary MN has changed diagnostic work-up and disease classification [2], and PLA2R status is now routinely assessed in both tissue and serum [3, 4].

Serum anti-PLA2R antibodies and glomerular PLA2R deposits provide complementary diagnostic information [5, 6]. Tissue staining is particularly useful in patients with nephrotic syndrome when serum is unavailable; serum anti-PLA2R antibodies are below detection, or a kidney biopsy is required for differential diagnosis. Reported diagnostic performance of PLA2R IHC varies considerably across cohorts and laboratories, reflecting differences in protocols, scoring systems, and cut-offs for PLA2R IHC positivity [4, 5, 7–18]. Consequently, laboratories commonly rely on local experience without formal validation. Sensitivity estimates reported in the literature range widely from 51% to 96%, underscoring the need for local laboratory validation. This study aims to address these sources of variation by comparing diagnostic performance and inter-observer reproducibility across two routine protocols and multiple cut-offs.

Frameworks such as intraclass correlation coefficients (ICC) and the Observers Needed to Evaluate Subjective Tests (ONEST) provide complementary approaches to reproducibility assessment [4, 17, 19, 20]. ICC quantifies agreement for the ordinal 0–3 intensity score, whereas the classic ONEST approach describes how the proportion of cases with identical (unanimous) dichotomized classifications changes as the number of observers (k) increases. Youden’s J index presents a straightforward method for identifying test-specific optimal cut-offs [21].

We conducted a multi-laboratory blinded observer study to examine diagnostic performance and inter-observer reproducibility of PLA2R IHC across two routine laboratory protocols. Rather than identifying a superior protocol, the primary aim was to examine how diagnostic accuracy and reproducibility interact across cut-offs, using Youden’s J and ONEST, and to propose a practical validation framework applicable to any laboratory implementing PLA2R IHC.

Our aims were to quantify diagnostic performance across cut-offs, assess inter-observer reliability and classification stability using ICC and ONEST, and examine the implications of any divergence between the statistically optimal cut-off and the most reproducible threshold for local cut-off selection.

## Methods

### Study design and reporting

We performed a retrospective diagnostic accuracy study comparing two routine laboratory protocols and reported according to STARD 2015 [22].

### Participants and reference standard

Two groups of adult native kidney biopsies were identified retrospectively. Membranous nephropathy (MN) cases were identified in the Danish Pathology and Genetics Data Bank, a nationwide registry in which all pathology specimens are coded according to the Danish SNOMED classification, using the morphology code M68130 (membranous glomerulonephritis) assigned by the reporting renal pathologist at the time of diagnosis. Eligible MN cases were native kidney biopsies performed at AUH between September 13, 2011, and February 22, 2021 (*n* = 50). Control biopsies were identified separately and comprised day-zero transplant biopsies and native kidney biopsies with non-MN glomerular diagnoses, selected by a renal pathologist in 2022 (*n* = 20). Controls were not retrieved through the M68130 search. Two of the 50 MN-coded biopsies were reclassified as ANCA-associated vasculitis and acute tubulointerstitial nephritis after review of electron microscopy and immunofluorescence and were therefore analyzed in the control group.

Of the 70 biopsies assessed for eligibility, 53 met the inclusion criteria (34 MN cases and 19 controls). Of these, 47 were assessable under both protocols and constituted the study population for the direct protocol comparison (20 pMN, 10 sMN, 17 controls). The flow of biopsies through the study, with reasons for exclusion at each step, is shown in Supplementary Figure S2.

Clinical primary MN (pMN) was defined as MN without a secondary cause after standard evaluation according to KDIGO 2021 guideline [1]. Secondary MN was defined by the presence of malignancy (within one year of biopsy), autoimmune disease (systemic lupus erythematosus, Sjögren’s syndrome, or systemic sclerosis), active infection, or exposure to known toxic medications. Classification as primary or secondary MN was based on review of the medical record and was performed independently of, and blinded to, the PLA2R IHC results.

The target condition for diagnostic analyses was PLA2R-associated membranous nephropathy. There is no independent gold standard. We used clinically defined primary MN (pMN) as the reference standard (disease-positive), and secondary MN (sMN) plus controls as disease-negative. Using clinically defined pMN carries a risk of misclassification because other target antigens may be present. However, it is a practical approach for assessing diagnostic tests when a true gold standard is absent. Because some clinically defined pMN cases are PLA2R-negative due to other antigens, PLA2R-based tests are not expected to achieve 100% sensitivity.

### Index test: PLA2R immunohistochemistry

PLA2R immunohistochemistry was not part of the routine diagnostic panel during the inclusion period, and any staining performed at diagnosis was not retrieved for comparison with the staining performed for this study. All staining was therefore performed for the purpose of this study on sections cut from archived FFPE blocks. MN cases were stained from blocks collected between 2011 and 2021; control biopsies were stained from more recently collected blocks (2015–2022), with tissue age at staining ranging from 7.6 to 91.6 months. Both protocols were applied to identical tissue blocks. Slides from each protocol were digitized immediately after staining, so observer assessment was performed on freshly prepared images for both protocols. However, the two staining runs were performed approximately 24 months apart, and the resulting difference in archival tissue age at the time of staining is addressed as a limitation.

The AUH protocol used the Ventana Benchmark Ultra platform with monoclonal antibody CL0474 at a 1:10,000 dilution, combined with high-pH retrieval (CC1) and OptiView DAB detection. In contrast, the HEH protocol used the Agilent Omnis platform, EnVision FLEX+ detection with linker amplification, and the same monoclonal antibody clone CL0474 (AMAb90772) at a 1:50,000 dilution. Notably, the AUH protocol used a higher antibody concentration than HEH (1:10,000 vs. 1:50,000; HEH was 5-fold more diluted), which may contribute to differences in background staining and optimal cut-off selection. These variations underscore the importance of careful consideration when adapting protocols to ensure consistency. Table 1 summarizes key protocol parameters that should be reported and locally validated to support reproducibility and comparability across laboratories.

**Table 1:** Proposed framework for local validation of PLA2R immunohistochemistry protocols

Parameter	AUH Protocol	HEH Protocol	Reporting and local validation considerations
Antibody Clone	CL0474	CL0474/AMAb90772	Report antibody clones and vendors; use a monoclonal antibody with documented performance.
Antibody Dilution	1:10,000	1:50,000	Higher dilution reduced background staining and improved single-observer reproducibility in our comparison. Dilution should be optimized locally together with cut-off selection.
Staining Platform	Ventana Benchmark Ultra	Agilent Omnis	Report platform and detection system in sufficient detail to enable reproducibility; protocol differences may influence background staining and optimal cut-offs.
Detection System	Ventana OptiView kit, DAB	EnVision FLEX+ (GV800) with mouse linker amplification	Use manufacturer-detection systems. Report detection kit and any amplification steps.
Antigen Retrieval	CC1 (Ventana), pH 8.1, 24 min	High-pH standard, 30 min (EnVision FLEX + kit GV800)	Report method, buffer pH, temperature, and duration. Retrieval conditions affect antigen accessibility and background staining.
Primary Antibody Incubation	16 min	20 min	Report on incubation time and temperature. Standardized incubation conditions reduce inter-run variability.
QC Procedures	On-slide internal control: normal kidney tissue with weak positive staining in podocytes	On-slide internal control: normal kidney tissue with weak positive staining in podocytes	Include positive and negative controls on each run. Day-zero transplant biopsies are useful for negative controls for background assessment.
Scoring Scale	0–3	0–3	0 = negative, 1 = weak, 2 = moderate, 3 = strong granular capillary wall staining. Non-granular patterns (e.g. isolated podocyte staining) are non-specific.
Recommended Cut-off (routine use)	≥ 3	≥ 1	Determine locally using Youden’s J index and ONEST to balance diagnostic accuracy and reproducibility. Optimal cut-off differed between protocols in our study, reflecting protocol-specific background staining.
Validation Controls	pMN, sMN, day-zero transplant biopsies, non-MN glomerulopathies	pMN, sMN, day-zero transplant biopsies, non-MN glomerulopathies	Include controls that probe background staining and specificity. Ideally, controls and MN cases should be stained from recent FFPE blocks to reflect routine diagnostic conditions.

This table is intended as a practical reporting and validation aid, not as a formal guideline. AUH: Aarhus University Hospital. HEH: Herlev Hospital. DAB: 3,3’-Diaminobenzidine. pMN: primary membranous nephropathy. sMN: secondary membranous nephropathy. FFPE: formalin-fixed paraffin-embedded. QC: Quality Control. CC1: Cell Conditioning 1 (Ventana high-pH retrieval buffer)

Seven observers, blinded to clinical data and diagnosis, independently scored granular capillary-wall staining on a 0–3 scale (0: no granular capillary-wall staining; 1: weak/sporadic; 2: moderate; 3: strong). Each individual rating assigned by an observer to a biopsy sample constituted an observer’s score. Non-granular patterns, such as isolated podocyte cytoplasmic staining, were considered non-specific. Observers recorded a biopsy as not assessable when the section contained insufficient glomeruli for evaluation; such ratings were treated as missing. For consensus analyses (Majority vote), a biopsy was considered positive if at least half of the observers scored at or above the selected cut-off, thus generating the consensus classification for each patient. Representative examples of PLA2R IHC scores 0–3 for both protocols are shown in Supplementary Figure S1.

### Serum anti-PLA2R antibodies

Serum anti-PLA2R antibodies (EUROIMMUN indirect immunofluorescence) were recorded when measured within ± 4 months of biopsy. Positivity was defined as a serum dilution titer of ≥ 1:10.

### Outcomes and statistical analysis

Primary outcomes were the sensitivity and specificity of PLA2R IHC, evaluated at a prespecified cut-off of ≥ 2. The optimal diagnostic cut-off was determined by maximizing Youden’s J index [21] (J = sensitivity + specificity − 1) across the evaluated thresholds (≥ 1, ≥ 2, ≥3).

Secondary outcomes included inter-observer agreement and diagnostic stability with increasing numbers of observers.

Sensitivity, specificity, positive predictive value (PPV), and negative predictive value (NPV) were calculated from 2 × 2 contingency tables at consensus level (majority vote, ≥ 4/7 observers positive) with 95% confidence intervals using Clopper-Pearson. Inter-observer agreement for the 0–3 score was quantified using a two-way random-effects ICC model to assess absolute agreement. ICC (2,1) estimates the expected reliability of a single rating drawn from the full rater pool, estimated simultaneously from all seven observers; it is therefore a panel-derived estimate and does not reflect the performance of any individual observer in isolation. ICC (2,7) estimates the reliability of the mean rating across all seven observers [20]. Reproducibility of dichotomized classifications with increasing numbers of observers was examined using the classic ONEST framework [19], which describes how overall percent agreement (OPA) changes as the number of observers (k) increases. OPA is expected to decline as k increases and approach a plateau when the panel size is sufficient. We summarized OPA across permutations (mean and 5th–95th percentile band) and visualized OPA as a function of k.

ICC and ONEST were calculated on complete cases, that is, biopsies scored by all seven observers (AUH n = 43, HEH n = 42). Biopsies with one or more not assessable ratings were excluded because both methods require a complete observer matrix. Diagnostic performance in Table 2 was calculated at consensus level and therefore includes all 47 patients. Weighted kappa was not calculated, as ONEST captures agreement dynamics across all panel sizes and is more informative for dichotomous classifications with multiple observers.

Analyses were performed in R (version 4.6.1).

## Results

### Participant characteristics

Patients with pMN were younger (median 62 years, IQR 57–71) than patients with sMN (median 67 years, IQR 56–76) and more often male (62% vs. 42%). Controls included IgA nephropathy (*n* = 2), ANCA-associated vasculitis (*n* = 3), acute tubulointerstitial nephritis (*n* = 1), focal segmental glomerulosclerosis (*n* = 1), and day-zero transplant biopsies (*n* = 10).

### PLA2R IHC score distribution and serology

**Fig. 1:**
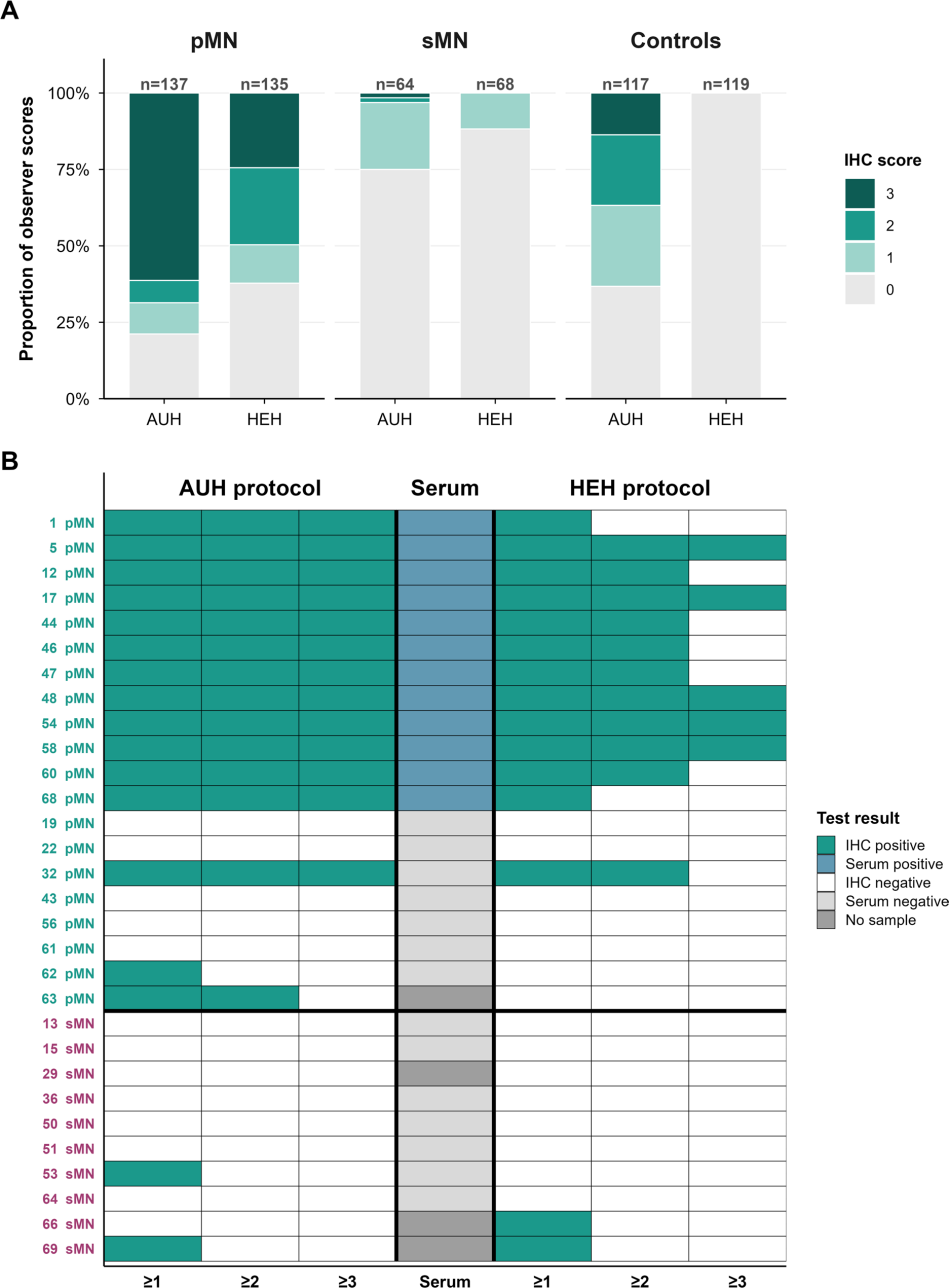
Distribution of PLA2R IHC scores and patient-level test results by staining protocols. **(A)** Distribution of individual observer PLA2R immunohistochemistry (PLA2R IHC) scores (0–3) across diagnostic groups (primary MN [pMN], secondary MN [sMN], and controls) for each staining protocol (AUH and HEH). Each bar shows the proportion of all individual observer scores within that group, so the unit of analysis is the single observer rating rather than the patient. Colour denotes IHC score (0, light grey; 1–3, increasing intensity of teal). Numbers above bars (n) indicate the total number of assessable observer scores per group. **(B)** Patient-level heatmap of dichotomized PLA2R IHC results and serum anti-PLA2R status. In contrast to **(A)**, each cell reflects the consensus classification for one patient (majority vote, ≥ 4/7 observers positive) rather than an individual observer score. Each row represents one patient (MN cases only; control biopsies are not shown); the row number is the patient identifier and the label colour denotes MN type (teal, pMN; magenta, sMN). Within each protocol (AUH, left block; HEH, right block), the three columns labelled ≥ 1, ≥2 and ≥ 3 indicate whether the consensus classification reached each positivity cut-off (teal, IHC positive; white, IHC negative). The central Serum column shows serum anti-PLA2R antibody status (blue, positive; light grey, negative). Grey cells indicate no available sample or non-assessable section. AUH, Aarhus University Hospital protocol; HEH, Herlev Hospital protocol

The distribution of individual observer PLA2R-IHC scores is shown in Fig. 1A. All seven observers assessed the same set of slides at each center. Of 329 possible observer scores per protocol (47 biopsies × 7 observers), 318 were obtained under the AUH protocol and 322 under the HEH protocol, giving 640 scores in total. The 11 and 7 missing ratings, respectively, were distributed across eight biopsies and were recorded as not assessable because the section contained insufficient glomeruli for evaluation, rather than reflecting any difference in the material examined.

At consensus level, most pMN cases were positive at the prespecified cut-off (≥ 2). The protocol-specific optimal cut-offs were ≥ 3 for AUH and ≥ 1 for HEH (Fig. 1B). All sMN cases were negative at ≥ 2 in both protocols; applying the lower HEH cut-off (≥ 1) introduced weak positivity in two sMN cases.

For the AUH protocol, 8/17 controls (47%) were positive at consensus level at ≥ 2, comprising seven day-zero transplant biopsies and one case of IgA nephropathy; all controls were negative under the HEH protocol. An inverse relationship between tissue age and false-positive rate was suggested: 6/9 controls at 7.6 months were false-positive (67%), compared with 2/6 at 19.6 months (33%) and 0/2 at 79.6–91.6 months (0%).

### Diagnostic performance

**Fig. 2:**
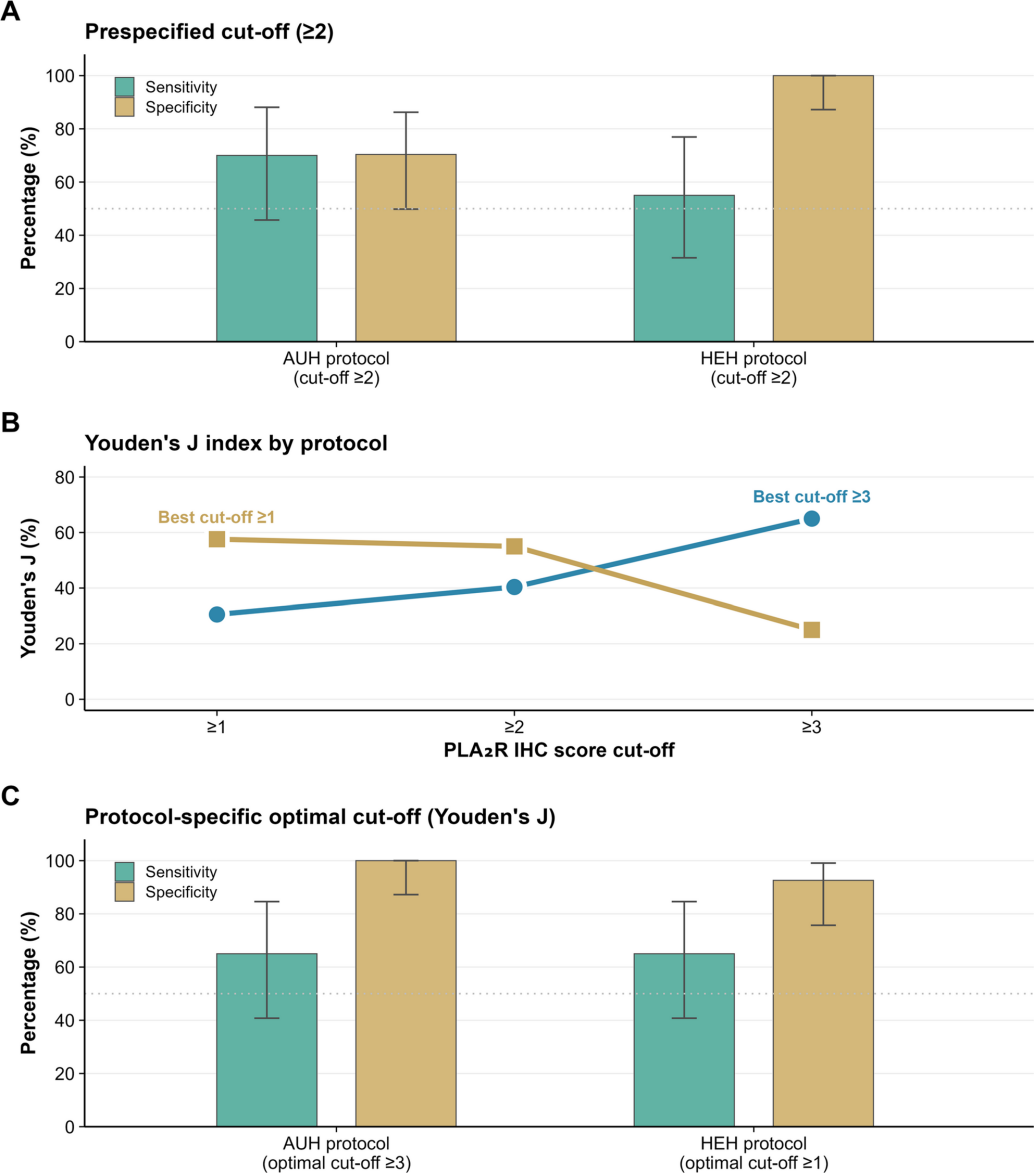
Diagnostic performance of PLA2R IHC across staining protocols and cut-offs. **(A)** Sensitivity and specificity (95% confidence intervals) using the prespecified positivity cut-off (≥ 2) for each protocol. **(B)** Youden’s J index across cut-offs (≥ 1, ≥ 2, ≥3) for each protocol, identifying the protocol-specific optimal threshold (AUH, ≥ 3; HEH, ≥ 1). **(C)** Sensitivity and specificity (95% confidence intervals) at the protocol-specific optimal cut-off. The dotted horizontal line in (A) and (C) marks 50%; error bars represent 95% confidence intervals

At the prespecified cut-off (≥ 2), consensus PLA2R IHC sensitivity and specificity were 70% (95% CI 46–88) and 70% (95% CI 50–86) for the AUH protocol and 55% (95% CI 32–77) and 100% (95% CI 87–100) for the HEH protocol, as shown in Fig. 2A.

Youden’s J indicated an optimal cut-off of 3 for the AUH protocol and 1 for the HEH protocol (Fig. 2B). Using these cut-offs, sensitivity/specificity was 65% (95% CI 40.8–84.6)/100% (95% CI 87.2–100.0) for AUH and 65% (95% CI 40.8–84.6)/92.6% (95% CI 75.7–99.1) for HEH, eliminating all false-positive controls, as shown in Fig. 2C. Diagnostic performance at all evaluated cut-offs is summarized in Table 2. Observer-level and patient-level diagnostic performance across all cut-offs are detailed in Supplementary Tables S2 and S3.

**Table 2:** Diagnostic performance of PLA2R IHC across cut-offs

Protocol	Cut-off	Sensitivity % (95% CI)	Specificity % (95% CI)	PPV % (95% CI)	NPV % (95% CI)	Youden’s J (%)
AUH	≥ 1	75 (51–91)	56 (35–75)	56 (35–75)	75 (51–91)	31
AUH	≥ 2	70 (46–88)	70 (50–86)	64 (41–83)	76 (55–91)	40
AUH	≥ 3 †	65 (41–85)	100 (87–100)	100 (75–100)	79 (62–91)	65
HEH	≥ 1 †	65 (41–85)	93 (76–99)	87 (60–98)	78 (60–91)	58
HEH	≥ 2	55 (32–77)	100 (87–100)	100 (72–100)	75 (58–88)	55
HEH	≥ 3	25 (9–49)	100 (87–100)	100 (48–100)	64 (48–78)	25

† Recommended cut-off for routine clinical use based on joint evaluation of Youden’s J and ONEST. All metrics calculated at consensus level (majority vote, ≥ 4/7 observers positive) with 95% CIs using Clopper-Pearson. *n* = 47 patients (20 pMN, 10 sMN, 17 controls). AUH and HEH protocols were applied to identical tissue blocks stained in August 2022 and June 2024, respectively; the systematic two-year difference in tissue age limits direct protocol comparison. PPV: positive predictive value. NPV: negative predictive value

### Inter-observer agreement and diagnostic stability

**Fig. 3:**
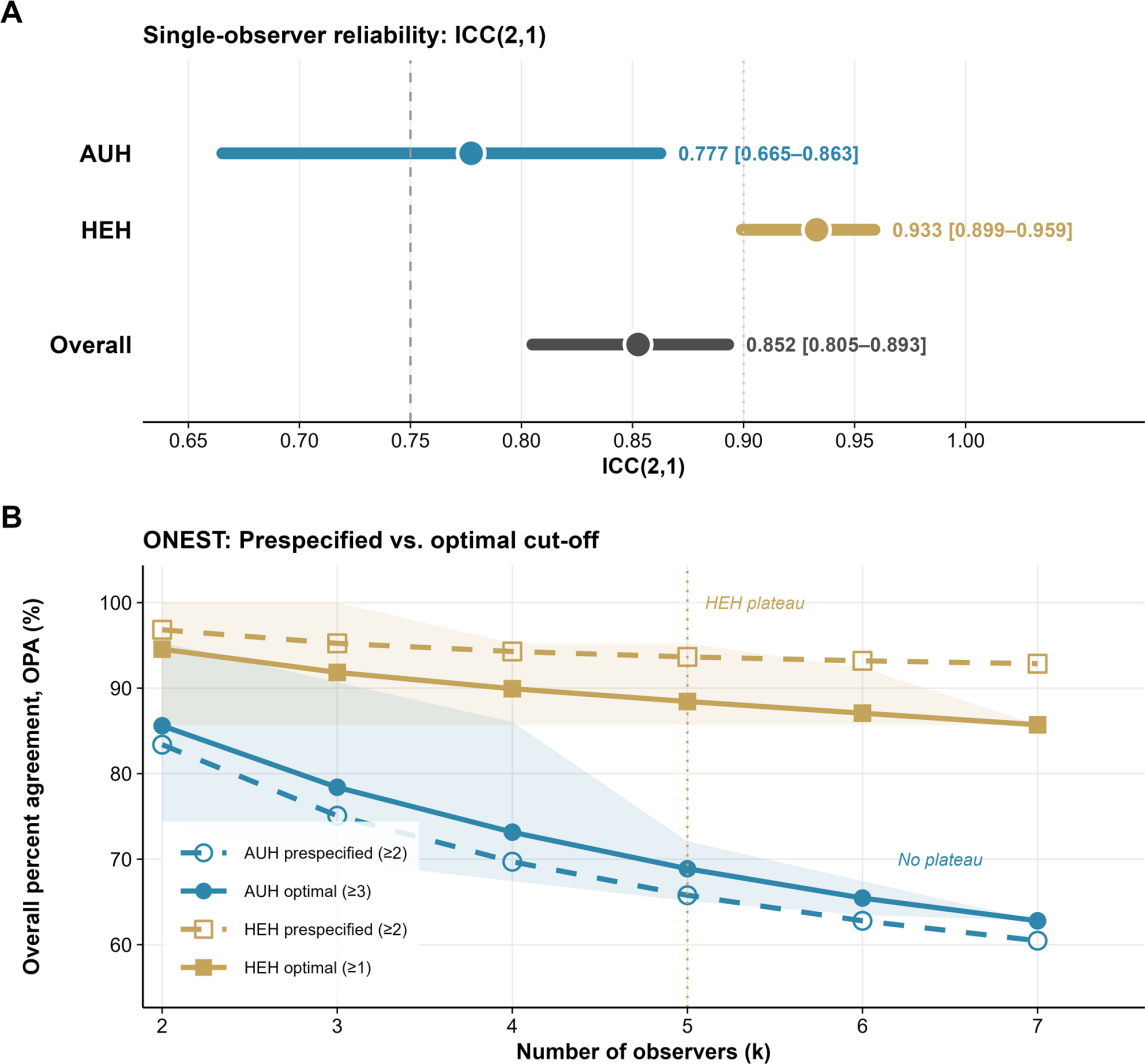
Inter-observer agreement and diagnostic stability of PLA2R IHC scoring. **(A)** Intraclass correlation coefficients ICC (2,1) with 95% confidence intervals for inter-observer agreement within each protocol (AUH and HEH) and overall. Dashed and dotted vertical lines indicate thresholds for good (0.75) and excellent (0.90) agreement, respectively. **(B)** ONEST analysis showing overall percent agreement (OPA) as a function of the number of observers (k = 2–7) at prespecified (≥ 2) and protocol-specific optimal cut-offs. Curves represent mean OPA across all possible observer combinations; shaded areas indicate the 5th–95th percentile range (optimal cut-offs only)

Inter-observer agreement and ONEST analyses for PLA2R IHC scoring are presented in Fig. 3.

As shown in Fig. 3A, the expected reliability of a single rating drawn from the rater pool was good overall [ICC (2,1) = 0.85 (95% CI: 0.80–0.89)], with excellent agreement for HEH [ICC (2,1) = 0.93 (0.90–0.96)] and good agreement for AUH [ICC (2,1) = 0.78 (0.67–0.86)]. The seven-observer panel demonstrated excellent reliability overall [ICC (2,7) = 0.96–0.99]. Inter-observer reproducibility metrics at each cut-off are summarized in Table 3.  

**Table 3:** Inter-observer reproducibility of PLA2R IHC across cut-offs

Protocol	Cut-off	ICC (2,1) [95% CI]	ICC (2,7)	OPA k = 7 (%)	ONEST Bandwidth
AUH	≥ 2	0.777 [0.665–0.863]	0.961	60	0.326
AUH	≥ 3 †	0.777 [0.665–0.863]	0.961	63	0.233
HEH	≥ 1 †	0.933 [0.899–0.959]	0.990	86	0.143
HEH	≥ 2	0.933 [0.899–0.959]	0.990	93	0.048

† Recommended cut-off for routine clinical use. ICC (2,1): expected reliability of a single randomly selected observer, estimated from all seven observers under a two-way random effects model. ICC (2,7): reliability of the seven-observer panel mean. OPA: overall percent agreement (unanimous agreement, k = 7). Bandwidth: maximum difference in OPA between best and worst observer pair combinations. ICC and ONEST were calculated on complete cases (AUH n = 43, HEH n = 42); biopsies with one or more not assessable ratings were excluded. A lower value indicates more stable reproducibility across observer pairs. AUH and HEH protocols were applied to identical tissue blocks stained in August 2022 and June 2024, respectively; the systematic two-year difference in tissue age limits direct protocol comparison

ONEST analyses showed the expected decline in OPA (unanimous agreement) with increasing numbers of observers (Fig. 3B). At the prespecified cut-off (≥ 2), OPA was higher for HEH (93%) than AUH (60%), indicating higher unanimity under the HEH protocol at this threshold. When comparing prespecified versus protocol-specific optimal cut-offs, HEH demonstrated higher unanimity at the prespecified cut-off (≥ 2; OPA 93%) than at the statistically optimal cut-off (≥ 1; OPA 86%), whereas AUH showed similar reproducibility at both cut-offs (≥ 2: 60%; ≥3: 63%). Complete patient-level scores are provided in Supplementary Table S4.

For the HEH protocol, ONEST curves stabilized beyond k = 5, with successive changes in OPA at the prespecified cut-off below 0.5 percentage points (pp), indicating that a seven-observer panel was sufficient for stable classification. For the AUH protocol, OPA continued to decline across panel sizes (ΔOPA ranging from − 8.3 to −2.3 pp), reflecting greater variability between observers and continued sensitivity to panel size. Bandwidth was narrower for HEH (0.048 at ≥ 2) than AUH (0.326 at ≥ 2), indicating less variation between observer pairs.

## Discussion

### Principal findings

This study describes the diagnostic performance and inter-observer reproducibility of two routine PLA2R IHC protocols and demonstrates that locally validated cut-offs can support clinically useful diagnostic performance for PLA2R-associated MN. Diagnostic accuracy and inter-observer reproducibility represent distinct performance dimensions that are not necessarily optimized at the same threshold. For the HEH protocol, the prespecified cut-off (≥ 2) yielded superior reproducibility compared to the statistically optimal cut-off (≥ 1) by Youden’s J, illustrating that both dimensions must be assessed during local protocol validation. Conclusions regarding protocol performance should be qualified by the specific cut-off applied, the tissue-handling conditions used, and the systematic difference in staining dates between protocols, which precludes unconfounded direct comparison.

### Diagnostic performance and validation

PLA2R IHC and serum anti-PLA2R antibodies provide concordant results in most cases. All seropositive patients were IHC-positive under both protocols at the recommended cut-offs; one patient was IHC-positive but seronegative, consistent with previous reports and supporting the complementary diagnostic role of tissue and serological testing. The diagnostic performance observed in our cohort is consistent with the published literature (sensitivity 51–96%, specificity 83–100%; Supplementary Table S1), despite the use of an unselected cohort without case enrichment for PLA2R-positive MN.

### Reference standard limitations

Clinically defined primary MN (pMN) was used as the reference standard in the absence of a histological or molecular gold standard. Primary MN is now recognized as antigenically heterogeneous: in European and North American cohorts, PLA2R accounts for approximately 70–80% of cases, while more than 20 additional target antigens including NELL-1, THSD7A, EXT1/EXT2, and others have been identified [6, 23]. A PLA2R-based assay will therefore inherently not achieve 100% sensitivity in an unselected pMN cohort. Sensitivity estimates should be interpreted as reflecting the proportion of clinically defined pMN cases with detectable PLA2R antigen deposition, rather than the intrinsic diagnostic sensitivity of the assay for PLA2R-associated MN specifically. LMD/MS would represent a more precise reference standard, as it identifies PLA2R-positive cases missed by conventional IHC and IF due to limited epitope accessibility [24], but was beyond the scope of this retrospective study.

### Protocol-specific background staining

Background staining and reproducibility differed between protocols, although the systematic two-year difference in staining dates precludes definitive attribution to antibody dilution alone. With this caveat, the HEH protocol showed superior inter-observer agreement and no false-positive controls, whereas the AUH protocol showed higher sensitivity at the prespecified cut-off but greater background staining. Protocol-specific cut-off optimization yielded comparable sensitivity and high specificity across both protocols.

All false-positive controls occurred under the AUH protocol, despite both protocols being applied to identical tissue blocks. Antibody concentration is a likely contributor: the five-fold lower concentration of the HEH protocol provides sufficient robustness to absorb background signal from recent FFPE tissue, whereas the higher AUH concentration makes the protocol more vulnerable. The false-positive rate rose with decreasing tissue age, with false-positive controls trending towards younger tissue than true-negative controls (median 7.6 vs. 19.6 months; W = 53, *p* = 0.078). These findings reinforce the need for protocol-specific validation using control material that reflects routine diagnostic practice.

### Inter-observer reliability and classification reproducibility

ICC and ONEST quantify distinct aspects of reproducibility. ICC measures inter-rater reliability for the ordinal 0–3 scale, independent of any cut-off. Single-rating reliability was good-to-excellent overall, and the seven-observer panel demonstrated excellent reliability.

High inter-rater reliability on the ordinal scale does not guarantee concordance in the dichotomous clinical classification: two observers assigning scores of 2 and 3 respectively contribute minimally to ICC disagreement yet reach opposite conclusions at cut-off ≥ 3. ONEST addresses this by quantifying the proportion of biopsies with identical positive/negative classifications at a specified cut-off, tracking how this changes as panel size increases from k = 2 to k = 7, and thereby identifying the minimum number of observers required for stable classification, that is, the panel size at which OPA reaches a plateau. The two frameworks are complementary: ICC confirms that the scoring system is applied consistently, providing the basis for reliable dichotomization, while ONEST determines whether consistent scoring translates into stable clinical decisions.

For HEH, the gap between single-observer and panel-level performance was small [ICC (2,1) = 0.933; OPA k = 7 = 93%; at cut-off ≥ 2; ΔOPA k = 6→7 = − 0.3 pp], indicating that a single observer performs comparably to the full panel. For AUH, the gap was substantially larger [ICC (2,1) = 0.777; OPA k = 7 = 60% at cut-off ≥ 2; ΔOPA k = 6→7 = − 2.3 pp]: consensus had not stabilized within the seven-observer panel, so the reported OPA of 60% likely overestimates true single-observer reproducibility. Applying cut-off ≥ 3 yielded only marginally higher OPA (63%), indicating that this reproducibility limitation reflects protocol characteristics rather than cut-off selection and cannot be addressed without reducing antibody concentration. The AUH laboratory has accordingly initiated a protocol revision; until implemented, cut-off ≥ 3 is recommended, as it eliminates false-positive controls and reduces ONEST bandwidth (0.233 vs. 0.326 at ≥ 2). Future studies should prospectively validate the revised AUH protocol, including PLA2R-positive MN cases stained from recent FFPE blocks to reflect routine diagnostic conditions.

### Cut-off selection: balancing accuracy and reproducibility

For the HEH protocol, the statistically optimal cut-off (Youden’s J) and the most reproducible threshold (ONEST) did not coincide. Cut-off ≥ 1 is recommended for routine clinical use, as the gain in sensitivity outweighs the modest reduction in reproducibility. The low antibody concentration of the HEH protocol substantially reduces background staining, supporting the interpretation that weak positive scores at ≥ 1 reflect true antigen deposition rather than non-specific signal. Residual specificity remains high. Cut-off selection therefore requires joint consideration of diagnostic accuracy, reproducibility, and clinical context, as outlined in Table 4.

**Table 4:** Proposed stepwise framework for cut-off selection in local PLA2R IHC validation

Step	Purpose	Metric	Implementation
1	Measure diagnostic performance	Sensitivity, Specificity, PPV, NPV	Calculate at all relevant cut-offs (≥ 1, ≥ 2, ≥3) using majority vote consensus. Report with 95% CIs.
2	Identify statistically optimal threshold	Youden’s J index	Select the cut-off maximizing J = sensitivity + specificity − 1. This represents the statistically optimal balance between sensitivity and specificity.
3	Evaluate ordinal scoring reliability	ICC (2,1) and ICC (2,7)	ICC (2,1) estimates single-observer reliability; ICC (2,7) estimates panel reliability. Both derived from all available observers under a two-way random effects model.
4	Evaluate classification stability	ONEST (OPA as function of k)	Determine the minimum number of observers for stable positive/negative classifications. Identify whether OPA reaches a plateau. If not, the protocol may require optimization.
5	Select final clinical cut-off	Joint evaluation of Youden’s J and ONEST	The statistically optimal cut-off (Youden’s J) and the most reproducible threshold (ONEST) may differ. When they diverge, cut-off selection should reflect the relative clinical cost of reduced sensitivity versus reduced reproducibility.

This framework is applicable to any semi-quantitative IHC scoring system where dichotomous clinical decisions are derived from ordinal scores. ICC: Intraclass correlation coefficient. ONEST: observers needed to evaluate subjective tests. OPA: overall percent agreement. PPV: positive predictive value. NPV: negative predictive value. CI: confidence interval

### Strengths and limitations

Strengths include blinded independent scoring by seven observers across two routine laboratory protocols in a real-world diagnostic cohort that was not enriched for PLA2R-positive cases, supporting the generalizability of the findings. Diagnostic performance was evaluated at both single-rating and panel level, with both prespecified and protocol-specific optimal cut-offs, using complementary reproducibility frameworks (ICC and ONEST). A stepwise validation framework is proposed (Table 4) that is generalizable beyond the two specific protocols examined. All diagnostic analyses were restricted to the 47 patients with material available for direct protocol comparison, ensuring methodological consistency.

Limitations include the retrospective design, modest sample size (*n* = 47), limited demographic diversity, and reliance on clinical classification as the reference standard. A critical limitation is that all 47 biopsies were stained under the AUH protocol in August 2022 and under the HEH protocol in June 2024, representing a systematic difference in tissue age of approximately two years across all biopsies. Observed differences between protocols cannot therefore be unambiguously attributed to protocol design alone. Serum anti-PLA2R was not available for all patients, limiting serological concordance analysis to a subset of MN cases. Finally, because ICC and ONEST require a complete observer matrix, biopsies containing not assessable ratings were excluded from these analyses, which may modestly favour reproducibility estimates by removing the least assessable sections.

## Conclusion

PLA2R IHC provides clinically useful diagnostic information for PLA2R-associated MN, but performance and reproducibility are protocol-dependent and were not necessarily optimized at the same cut-off. Laboratories should therefore validate both dimensions locally rather than adopting a single fixed cut-off, and the stepwise framework proposed in Table 4 offers a practical guide for doing so. Future validation studies should apply both protocols to matched tissue under controlled conditions to allow unconfounded protocol comparison.

## Supplementary Information

Below is the link to the electronic supplementary material.

Supplementary Material 1

## Data Availability

The datasets used and/or analyzed during the current study are available from the corresponding author on reasonable request.

## Abbreviations

AUH: Aarhus University Hospital
HEH: Herlev Hospital
IHC: Immunohistochemistry
ICC: Intraclass correlation coefficient
MN: Membranous nephropathy
OPA: overall percent agreement
pMN: primary membranous nephropathy
sMN: secondary membranous nephropathy
ONEST: Observers needed to evaluate subjective tests
PLA2R: Phospholipase A2 receptor
