# Supplementary material for: Diagnostic performance and inter-observer reproducibility of glomerular PLA2R immunohistochemistry in primary membranous nephropathy: a multi-laboratory blinded observer study
Source: BMC Nephrol. 2026 Jul 28;27:467. doi: 10.1186/s12882-026-05233-0 (PMC13463983; doi:10.1186/s12882-026-05233-0)
Supplement: Supplementary file 1 [file 12882_2026_5233_MOESM1_ESM.docx]

**Figure S1.** **Illustrating the PLA2R IHC staining at center specific protocols**

*At AUH, Ventana Benchmark Ultra, monoclonal CL0474 (1:10,000), high-pH retrieval (CC1), OptiView DAB was used. At HEH using Agilent Omnis EnVision FLEX+, monoclonal CL0474 (1:50,000), high-pH retrieval, linker amplification.*

*The AUH staining shows a generally higher overall staining intensity with more diffuse background signal, making the transition from background only to weakly positive staining (score 1) less distinct, whereas the HEH staining has minimal background and sharply demarcated capillary wall staining, such that any visible signal is more readily classified as positive.*

**Figure S2. Flow of biopsies through the study**

*MN cases were identified in the Danish Pathology and Genetics Data Bank using SNOMED morphology code M68130; control biopsies were selected separately by a renal pathologist and were not retrieved through the M68130 search. Two MN-coded biopsies were reclassified after reviewing electron microscopy and immunofluorescence and were analyzed in the control group. Of 53 biopsies meeting the inclusion criteria, 47 were assessable under both protocols and formed the study population for the direct protocol comparison. Observer scores were recorded as not assessable when the section contained insufficient glomeruli for evaluation. AUH: Aarhus University Hospital. HEH: Herlev Hospital. IHC: immunohistochemistry. MN: Membranous Nephropathy. pMN: primary membranous nephropathy. sMN: secondary membranous nephropathy.*

**Table S1. Summary of published studies reporting PLA2R IHC sensitivity and specificity in membranous nephropathy.**

| Country | Population | Cut-off | Sensitivity (%) | Specificity (%) |
| --- | --- | --- | --- | --- |
| Germany | pMN=67; sMN=27 | Dichotomous | 69 | 100 |
| India | pMN=51; sMN=31; ctrl=13 | Dichotomous | 91.8 | 95.1 |
| Italy | pMN=72; sMN=23 | Dichotomous | 71 | 83 |
| Germany | MN=263; Ab+=167 | Dichotomous | NR | NR |
| India | pMN=99; sMN=54; ctrl=37 | ≥1+ | 70.2 | 96.6 |
| China | pMN=59; sMN=56 | ≥1+ | 83.1 | 87.5 |
| South Africa | pMN=41; lupus/DM=47 | Not stated | 51.2 | 100 |
| Iran | pMN=79; sMN=29 | ≈≥2+ | 96.2** | 97.1** |
| China | pMN=179; LN=40; HBV=26 | Not stated | 92.2 | 94.2 |
| Spain | pMN=47; sMN=17 | NA | 74.4 | 94.1 |
| Denmark | pMN=21; sMN=13; ctrl=19 | ≥3+ (Youden) | 65.0 | 100 |
| Denmark | pMN=20; sMN=13; ctrl=17 | ≥1+ (Youden) | 65.0 | 92.9 |

*IHC scoring system categories:*

- ***Dichotomous (positive/negative);*** *staining recorded as present/absent based on a predefined pattern (typically granular GBM staining without podocyte staining).*
- ***Semi-quantitative 0-3+*;*** *0 (negative), 1+ (weak), 2+ (moderate), 3+ (strong) glomerular capillary-wall staining; studies then apply a cut-off (most commonly ≥2+) to define a positive test.*

***For Afghani 2021, sensitivity and specificity refer to the combined PLA₂R+/IgG4+ definition, not PLA2R alone.*

*‡ Wiech et al. investigated the diagnostic role of renal biopsy in patients with nephrotic syndrome and circulating PLA2R antibodies. Their cohort comprised biopsy-proven MN enriched PLA2R-associated disease; PLA2R IHC was used to confirm PLA2R-associated MN and to detect additional diagnoses, rather than to distinguish primary from secondary MN in an unselected population. Formal sensitivity and specificity estimate for PLAR IHC vs secondary MN were therefore not reported.*

*Abbreviations: pMN, primary membranous nephropathy; sMN, secondary membranous nephropathy; ctrl, controls; LN, lupus nephritis; HBV, hepatitis B virus; NR, not reported; NA, not available*

**Table S2. Observer-level diagnostic performance of PLA2R IHC across cut-offs 1-3**

| **Cutoff** | **Center** | **Observer** | **Patients (n)** | **TP** | **FN** | **FP** | **TN** | **Sensitivity (95% CI)** | **Specificity (95% CI)** | **PPV** | **NPV** | **Youden's J (%)** |
| --- | --- | --- | --- | --- | --- | --- | --- | --- | --- | --- | --- | --- |
| Cut-off 1 | AUH | 1 | 47 | 17 | 3 | 15 | 12 | 85 (62–97) | 44 (26–65) | 53 | 80 | 29 |
| Cut-off 1 | AUH | 2 | 45 | 17 | 3 | 13 | 12 | 85 (62–97) | 48 (28–69) | 57 | 80 | 33 |
| Cut-off 1 | AUH | 3 | 45 | 13 | 6 | 7 | 19 | 68 (43–87) | 73 (52–88) | 65 | 76 | 42 |
| Cut-off 1 | AUH | 4 | 45 | 13 | 6 | 8 | 18 | 68 (43–87) | 69 (48–86) | 62 | 75 | 38 |
| Cut-off 1 | AUH | 5 | 45 | 16 | 3 | 14 | 12 | 84 (60–97) | 46 (27–67) | 53 | 80 | 30 |
| Cut-off 1 | AUH | 6 | 46 | 15 | 5 | 13 | 13 | 75 (51–91) | 50 (30–70) | 54 | 72 | 25 |
| Cut-off 1 | AUH | 7 | 45 | 17 | 3 | 20 | 5 | 85 (62–97) | 20 (7–41) | 46 | 62 | 5 |
| Cut-off 1 | HEH | 1 | 47 | 13 | 7 | 2 | 25 | 65 (41–85) | 93 (76–99) | 87 | 78 | 58 |
| Cut-off 1 | HEH | 2 | 47 | 13 | 7 | 2 | 25 | 65 (41–85) | 93 (76–99) | 87 | 78 | 58 |
| Cut-off 1 | HEH | 3 | 47 | 13 | 7 | 2 | 25 | 65 (41–85) | 93 (76–99) | 87 | 78 | 58 |
| Cut-off 1 | HEH | 4 | 47 | 13 | 7 | 2 | 25 | 65 (41–85) | 93 (76–99) | 87 | 78 | 58 |
| Cut-off 1 | HEH | 5 | 42 | 10 | 7 | 0 | 25 | 59 (33–82) | 100 (86–100) | 100 | 78 | 59 |
| Cut-off 1 | HEH | 6 | 45 | 11 | 7 | 0 | 27 | 61 (36–83) | 100 (87–100) | 100 | 79 | 61 |
| Cut-off 1 | HEH | 7 | 47 | 11 | 9 | 0 | 27 | 55 (32–77) | 100 (87–100) | 100 | 75 | 55 |
| Cut-off 2 | AUH | 1 | 47 | 14 | 6 | 8 | 19 | 70 (46–88) | 70 (50–86) | 64 | 76 | 40 |
| Cut-off 2 | AUH | 2 | 45 | 14 | 6 | 9 | 16 | 70 (46–88) | 64 (42–82) | 61 | 73 | 34 |
| Cut-off 2 | AUH | 3 | 45 | 12 | 7 | 0 | 26 | 63 (38–84) | 100 (87–100) | 100 | 79 | 63 |
| Cut-off 2 | AUH | 4 | 45 | 11 | 8 | 0 | 26 | 58 (34–80) | 100 (87–100) | 100 | 76 | 58 |
| Cut-off 2 | AUH | 5 | 45 | 14 | 5 | 10 | 16 | 74 (49–91) | 62 (41–80) | 58 | 76 | 35 |
| Cut-off 2 | AUH | 6 | 46 | 14 | 6 | 6 | 20 | 70 (46–88) | 77 (56–91) | 70 | 77 | 47 |
| Cut-off 2 | AUH | 7 | 45 | 15 | 5 | 12 | 13 | 75 (51–91) | 52 (31–72) | 56 | 72 | 27 |
| Cut-off 2 | HEH | 1 | 47 | 11 | 9 | 0 | 27 | 55 (32–77) | 100 (87–100) | 100 | 75 | 55 |
| Cut-off 2 | HEH | 2 | 47 | 11 | 9 | 0 | 27 | 55 (32–77) | 100 (87–100) | 100 | 75 | 55 |
| Cut-off 2 | HEH | 3 | 47 | 11 | 9 | 0 | 27 | 55 (32–77) | 100 (87–100) | 100 | 75 | 55 |
| Cut-off 2 | HEH | 4 | 47 | 11 | 9 | 0 | 27 | 55 (32–77) | 100 (87–100) | 100 | 75 | 55 |
| Cut-off 2 | HEH | 5 | 42 | 8 | 9 | 0 | 25 | 47 (23–72) | 100 (86–100) | 100 | 74 | 47 |
| Cut-off 2 | HEH | 6 | 45 | 7 | 11 | 0 | 27 | 39 (17–64) | 100 (87–100) | 100 | 71 | 39 |
| Cut-off 2 | HEH | 7 | 47 | 8 | 12 | 0 | 27 | 40 (19–64) | 100 (87–100) | 100 | 69 | 40 |
| Cut-off 3 | AUH | 1 | 47 | 13 | 7 | 1 | 26 | 65 (41–85) | 96 (81–100) | 93 | 79 | 61 |
| Cut-off 3 | AUH | 2 | 45 | 11 | 9 | 1 | 24 | 55 (32–77) | 96 (80–100) | 92 | 73 | 51 |
| Cut-off 3 | AUH | 3 | 45 | 11 | 8 | 0 | 26 | 58 (34–80) | 100 (87–100) | 100 | 76 | 58 |
| Cut-off 3 | AUH | 4 | 45 | 11 | 8 | 0 | 26 | 58 (34–80) | 100 (87–100) | 100 | 76 | 58 |
| Cut-off 3 | AUH | 5 | 45 | 14 | 5 | 8 | 18 | 74 (49–91) | 69 (48–86) | 64 | 78 | 43 |
| Cut-off 3 | AUH | 6 | 46 | 10 | 10 | 0 | 26 | 50 (27–73) | 100 (87–100) | 100 | 72 | 50 |
| Cut-off 3 | AUH | 7 | 45 | 14 | 6 | 7 | 18 | 70 (46–88) | 72 (51–88) | 67 | 75 | 42 |
| Cut-off 3 | HEH | 1 | 47 | 5 | 15 | 0 | 27 | 25 (9–49) | 100 (87–100) | 100 | 64 | 25 |
| Cut-off 3 | HEH | 2 | 47 | 5 | 15 | 0 | 27 | 25 (9–49) | 100 (87–100) | 100 | 64 | 25 |
| Cut-off 3 | HEH | 3 | 47 | 5 | 15 | 0 | 27 | 25 (9–49) | 100 (87–100) | 100 | 64 | 25 |
| Cut-off 3 | HEH | 4 | 47 | 5 | 15 | 0 | 27 | 25 (9–49) | 100 (87–100) | 100 | 64 | 25 |
| Cut-off 3 | HEH | 5 | 42 | 5 | 12 | 0 | 25 | 29 (10–56) | 100 (86–100) | 100 | 68 | 29 |
| Cut-off 3 | HEH | 6 | 45 | 3 | 15 | 0 | 27 | 17 (4–41) | 100 (87–100) | 100 | 64 | 17 |
| Cut-off 3 | HEH | 7 | 47 | 5 | 15 | 0 | 27 | 25 (9–49) | 100 (87–100) | 100 | 64 | 25 |

*Observer-level diagnostic accuracy of glomerular PLA₂R immunohistochemistry (IHC) at cut-offs ≥1, ≥2, and ≥3, stratified by center (AUH and HEH) and observer (1–7). For each combination of center, observer, and cut-off, the table shows the number of patients (n), true positives (TP), false negatives (FN), false positives (FP), true negatives (TN), and the resulting sensitivity, specificity, positive predictive value (PPV), negative predictive value (NPV), and Youden’s J. The reference standard is clinically defined primary membranous nephropathy (pMN), with secondary MN and controls classified as non-pMN.*

**Table S3. Patient-level diagnostic performance of PLA2R IHC and serum PLA2R by cut-off and center**

| **Modality** | **Cutoff** | **Center** | **TP** | **FP** | **FN** | **TN** | **Sensitivity** | **Specificity** | **PPV (%)** | **NPV (%)** | **Youden's J (%)** |
| --- | --- | --- | --- | --- | --- | --- | --- | --- | --- | --- | --- |
| IHC PLA2R | Cut-off 1 | AUH | 15 | 12 | 5 | 15 | 75 (51–91) | 56 (35–74) | 56 | 75 | 31 |
| IHC PLA2R | Cut-off 1 | HEH | 13 | 2 | 7 | 25 | 65 (41–85) | 93 (76–99) | 87 | 78 | 58 |
| IHC PLA2R | Cut-off 2 | AUH | 14 | 8 | 6 | 19 | 70 (46–88) | 70 (50–86) | 64 | 76 | 40 |
| IHC PLA2R | Cut-off 2 | HEH | 11 | 0 | 9 | 27 | 55 (32–77) | 100 (87–100) | 100 | 75 | 55 |
| IHC PLA2R | Cut-off 3 | AUH | 13 | 0 | 7 | 27 | 65 (41–85) | 100 (87–100) | 100 | 79 | 65 |
| IHC PLA2R | Cut-off 3 | HEH | 5 | 0 | 15 | 27 | 25 (9–49) | 100 (87–100) | 100 | 64 | 25 |
| Serum PLA2R | Serum | AUH | 12 | 0 | 7 | 7 | 63 (38–84) | 100 (59–100) |  |  | 63 |
| Serum PLA2R | Serum | HEH | 12 | 0 | 7 | 7 | 63 (38–84) | 100 (59–100) |  |  | 63 |

*Patient-level diagnostic accuracy of PLA2R immunohistochemistry (IHC) based on ≥50% of observers scoring at or above the given cut-off. Cut-offs ≥1, ≥2, and ≥3, and of serum PLA₂R, stratified by center (AUH and HEH) are shown. For each modality, cut-off, and center, the table reports true positives (TP), false positives (FP), false negatives (FN), true negatives (TN), sensitivity, specificity, positive predictive value (PPV), negative predictive value (NPV), and Youden’s J. The reference standard is clinically defined primary membranous nephropathy (pMN), with secondary MN and controls classified as non-pMN.*

**Table S4. Patient-level PLA₂R IHC scores by observer, center, serum status, and diagnosis**

| **Patient-ID** | Observer 1 (AUH) | Observer 2 (AUH) | Observer 3 (AUH) | Observer 4 (AUH) | Observer 5 (AUH) | Observer 6 (AUH) | Observer 7 (AUH) | Observer 1 (HEH) | Observer 2 (HEH) | Observer 3 (HEH) | Observer 4 (HEH) | Observer 5 (HEH) | Observer 6 (HEH) | Observer 7 (HEH) | Anti-PLAR-ab | MN type |
| --- | --- | --- | --- | --- | --- | --- | --- | --- | --- | --- | --- | --- | --- | --- | --- | --- |
| **1** | 3 | 2 | 3 | 3 | 3 | 2 | 3 | 1 | 1 | 1 | 1 | 1 | 1 | 0 | Pos. | Primary |
| **2** | 2 | 3 | 1 | 1 | 3 | 2 | 3 | 0 | 0 | 0 | 0 | 0 | 0 | 0 | NA | Control (day-zero biopsy) |
| **3** | No tissue | No tissue | No tissue | No tissue | No tissue | No tissue | No tissue | No tissue | No tissue | No tissue | No tissue | No tissue | No tissue | No tissue | NA | Biopsy not available |
| **4** | 2 | 2 | 1 | 1 | 3 | 2 | 3 | 0 | 0 | 0 | 0 | 0 | 0 | 0 | NA | Control (day-zero biopsy) |
| **5** | 3 | 3 | 3 | 3 | 3 | 3 | 3 | 3 | 3 | 3 | 3 | No IHC score | No IHC score | 3 | Pos. | Primary |
| **6** | No tissue | No tissue | No tissue | No tissue | No tissue | No tissue | No tissue | No tissue | No tissue | No tissue | No tissue | No tissue | No tissue | No tissue | NA | Biopsy not available |
| **7** | 0 | No IHC score | 0 | 0 | 0 | 0 | 0 | No IHC score | No IHC score | No IHC score | No IHC score | No IHC score | No IHC score | No IHC score | NA | Control (IgA nephropathy) |
| **8** | 1 | 2 | 0 | 1 | 2 | 2 | 3 | 0 | 0 | 0 | 0 | 0 | 0 | 0 | NA | Control (day-zero biopsy) |
| **9** | No tissue | No tissue | No tissue | No tissue | No tissue | No tissue | No tissue | No tissue | No tissue | No tissue | No tissue | No tissue | No tissue | No tissue | NA | Biopsy not available |
| **10** | 2 | 2 | 1 | 1 | 3 | 2 | 3 | 0 | 0 | 0 | 0 | 0 | 0 | 0 | NA | Control (day-zero biopsy) |
| **11** | No tissue | No tissue | No tissue | No tissue | No tissue | No tissue | No tissue | No tissue | No tissue | No tissue | No tissue | No tissue | No tissue | No tissue | NA | Biopsy not available |
| **12** | 3 | 3 | 3 | 3 | 3 | 2 | 3 | 2 | 2 | 2 | 2 | No IHC score | No IHC score | 1 | Pos. | Primary |
| **13** | 0 | 0 | 0 | 0 | 0 | 0 | 1 | 0 | 0 | 0 | 0 | 0 | 0 | 0 | Neg. | Secondary (pulmonary cancer) |
| **14** | No tissue | No tissue | No tissue | No tissue | No tissue | No tissue | No tissue | No tissue | No tissue | No tissue | No tissue | No tissue | No tissue | No tissue | NA | Biopsy not available |
| **15** | 1 | 0 | 0 | 0 | 0 | 0 | 1 | 0 | 0 | 0 | 0 | 0 | 0 | 0 | Neg. | Secondary (Sarcoidosis) |
| **16** | 1 | No IHC score | 0 | 0 | 0 | 1 | No IHC score | 0 | 0 | 0 | 0 | 0 | 0 | 0 | NA | Control (IgA nephropathy) |
| **17** | 3 | 3 | 3 | 3 | 3 | 3 | 3 | 3 | 3 | 3 | 3 | 2 | 2 | 2 | Pos. | Primary |
| **18** | 0 | 0 | 0 | 0 | 0 | 0 | 1 | 0 | 0 | 0 | 0 | 0 | 0 | 0 | NA | Control (day-zero biopsy) |
| **19** | 0 | 1 | 0 | 0 | 0 | 0 | 0 | 0 | 0 | 0 | 0 | 0 | 0 | 0 | Neg. | Primary |
| **20** | No tissue | No tissue | No tissue | No tissue | No tissue | No tissue | No tissue | No tissue | No tissue | No tissue | No tissue | No tissue | No tissue | No tissue | NA | Biopsy not available |
| **21** | 2 | 2 | 0 | 1 | 3 | 1 | 3 | 0 | 0 | 0 | 0 | 0 | 0 | 0 | NA | Control (IgA nephropathy) |
| **22** | 1 | 0 | 0 | 0 | 1 | 0 | 2 | 0 | 0 | 0 | 0 | 0 | 0 | 0 | Neg. | Primary |
| **23** | 2 | 2 | 1 | 1 | 3 | 2 | 3 | 0 | 0 | 0 | 0 | 0 | 0 | 0 | NA | Control (day-zero biopsy) |
| **24** | No tissue | No tissue | No tissue | No tissue | No tissue | No tissue | No tissue | No tissue | No tissue | No tissue | No tissue | No tissue | No tissue | No tissue | NA | Biopsy not available |
| **25** | No tiss | No tissue | No tissue | No tissue | No tissue | No tissue | No tissue | No tissue | No tissue | No tissue | No tissue | No tissue | No tissue | No tissue | NA | Biopsy not available |
| **26** | 1 | 1 | 0 | 0 | 1 | 1 | 1 | 0 | 0 | 0 | 0 | 0 | 0 | 0 | NA | Control (focal segmental glomerulosclerosis) |
| **27** | 0 | 1 | 0 | 0 | 1 | 0 | 2 | 0 | 0 | 0 | 0 | 0 | 0 | 0 | NA | Control (day-zero biopsy) |
| **28** | 2 | 2 | 0 | 0 | 3 | 1 | 2 | 0 | 0 | 0 | 0 | 0 | 0 | 0 | NA | Control (day-zero biopsy) |
| **29** | 0 | 0 | 0 | 0 | 1 | 0 | 1 | 0 | 0 | 0 | 0 | 0 | 0 | 0 | NA | Secondary (Lymphoma) |
| **30** | 1 | No IHC score | No IHC score | 0 | No IHC score | 0 | 1 | No IHC score | No IHC score | No IHC score | No IHC score | No IHC score | No IHC score | No IHC score | NA | Secondary (systemic lupus erythematosus) |
| **31** | 0 | 1 | 0 | 0 | 0 | 0 | 0 | 0 | 0 | 0 | 0 | 0 | 0 | 0 | Neg. | Excluded (No deposit on EM) |
| **32** | 3 | 2 | No IHC score | No IHC score | 3 | 3 | 3 | 2 | 2 | 2 | 2 | 3 | 2 | 2 | Neg. | Primary |
| **33** | No IHC score | No IHC score | No IHC score | No IHC score | No IHC score | No IHC score | No IHC score | 0 | 0 | 0 | 0 | 0 | 0 | 0 | Neg. | Secondary (autoimmune disease) |
| **34** | 0 | 0 | 0 | 0 | 1 | 0 | 2 | 0 | 0 | 0 | 0 | 0 | 0 | 0 | NA | Control (day-zero biopsy) |
| **35** | 2 | 2 | 1 | 1 | 3 | 2 | 3 | 0 | 0 | 0 | 0 | 0 | 0 | 0 | NA | Control (day-zero biopsy) |
| **36** | 0 | 1 | 0 | 0 | 3 | 0 | 0 | 0 | 0 | 0 | 0 | No IHC score | 0 | 0 | Neg. | Secondary (Scleroderma) |
| **37** | 0 | 0 | 0 | 0 | 0 | 0 | 1 | 0 | 0 | 0 | 0 | 0 | 0 | 0 | NA | Control (ANCA-associated vasculitis) |
| **38** | No tissue | No tissue | No tissue | No tissue | No tissue | No tissue | No tissue | No tissue | No tissue | No tissue | No tissue | No tissue | No tissue | No tissue | NA | Biopsy not available |
| **39** | No tissue | No tissue | No tissue | No tissue | No tissue | No tissue | No tissue | No tissue | No tissue | No tissue | No tissue | No tissue | No tissue | No tissue | NA | Biopsy not available |
| **40** | No tissue | No tissue | No tissue | No tissue | No tissue | No tissue | No tissue | No tissue | No tissue | No tissue | No tissue | No tissue | No tissue | No tissue | NA | Biopsy not available |
| **41** | 2 | 2 | 2 | 1 | 3 | 1 | 3 | No tissue | No tissue | No tissue | No tissue | No tissue | No tissue | No tissue | NA | Control (minimal change disease) |
| **42** | No tissue | No tissue | No tissue | No tissue | No tissue | No tissue | No tissue | No tissue | No tissue | No tissue | No tissue | No tissue | No tissue | No tissue | NA | Biopsy not available |
| **43** | 0 | 0 | 0 | 0 | 0 | 0 | 0 | 0 | 0 | 0 | 0 | 0 | 0 | 0 | Neg. | Primary |
| **44** | 3 | 3 | 3 | 3 | 3 | 3 | 3 | 2 | 2 | 2 | 2 | 1 | 1 | 0 | Pos. | Primary |
| **45** | 1 | 2 | 1 | 1 | 2 | 1 | 2 | 0 | 0 | 0 | 0 | 0 | 0 | 0 | NA | Control (ANCA-associated vasculitis) |
| **46** | 3 | 3 | 3 | 3 | 3 | 3 | 3 | 2 | 2 | 2 | 2 | 2 | 1 | 1 | Pos. | Primary |
| **47** | 3 | 3 | 3 | 3 | 3 | 3 | 3 | 2 | 2 | 2 | 2 | 3 | 2 | 2 | Pos. | Primary |
| **48** | 3 | 3 | 3 | 3 | 3 | 2 | 3 | 3 | 3 | 3 | 3 | 3 | 2 | 3 | Pos. | Primary |
| **49** | No IHC score | No IHC score | No IHC score | No IHC score | No IHC score | No IHC score | No IHC score | No IHC score | No IHC score | No IHC score | No IHC score | No IHC score | No IHC score | No IHC score | Neg. | Secondary (breast cancer) |
| **50** | 0 | 0 | 0 | 0 | 0 | 0 | 1 | 0 | 0 | 0 | 0 | 0 | 0 | 0 | Neg. | Secondary (graft-versus-host disease) |
| **51** | 0 | 0 | 0 | 0 | 0 | 0 | 0 | 0 | 0 | 0 | 0 | 0 | 0 | 0 | Neg. | Secondary (Lymphoma) |
| **52** | 0 | 0 | 0 | 0 | 0 | 0 | 0 | 0 | 0 | 0 | 0 | 0 | 0 | 0 | NA | Control (ANCA-associated vasculitis) |
| **53** | 1 | No IHC score | No IHC score | No IHC score | No IHC score | No IHC score | No IHC score | 0 | 0 | 0 | 0 | No IHC score | 0 | 0 | Neg. | Secondary (systemic lupus erythematosus) |
| **54** | 3 | 3 | 3 | 3 | 3 | 3 | 3 | 3 | 3 | 3 | 3 | 3 | 3 | 3 | Pos. | Primary |
| **55** | No tissue | No tissue | No tissue | No tissue | No tissue | No tissue | No tissue | No tissue | No tissue | No tissue | No tissue | No tissue | No tissue | No tissue | NA | Biopsy not available |
| **56** | 0 | 0 | 0 | 0 | 0 | 0 | 0 | 0 | 0 | 0 | 0 | 0 | 0 | 0 | Neg. | Primary |
| **57** | 3 | 0 | 0 | 0 | 0 | 0 | 0 | 0 | 0 | 0 | 0 | 0 | 0 | 0 | NA | Control (acute tubulointerstitial nephritis) |
| **58** | 3 | 3 | 3 | 3 | 3 | 3 | 3 | 3 | 3 | 3 | 3 | 3 | 3 | 3 | Pos. | Primary |
| **59** | No tissue | No tissue | No tissue | No tissue | No tissue | No tissue | No tissue | No tissue | No tissue | No tissue | No tissue | No tissue | No tissue | No tissue | NA | Biopsy not available |
| **60** | 3 | 3 | 2 | 3 | 3 | 3 | 3 | 2 | 2 | 2 | 2 | No IHC score | 3 | 3 | Pos. | Primary |
| **61** | 1 | 1 | 0 | 0 | No IHC score | 0 | 1 | 0 | 0 | 0 | 0 | 0 | 0 | 0 | Neg. | Primary |
| **62** | 1 | 1 | 0 | 0 | 1 | 1 | 1 | 0 | 0 | 0 | 0 | 0 | 0 | 0 | Neg. | Primary |
| **63** | 2 | 2 | 1 | 1 | 3 | 2 | 3 | 0 | 0 | 0 | 0 | 0 | 0 | 1 | Neg. | Primary |
| **64** | 0 | 1 | 0 | 0 | 0 | 1 | 2 | 0 | 0 | 0 | 0 | 0 | 0 | 0 | Neg. | Secondary (systemic lupus erythematosus) |
| **65** | 2 | 3 | 3 | 0 | 3 | 2 | 2 | No tissue | No tissue | No tissue | No tissue | No tissue | No tissue | No tissue | Pos. | Primary |
| **66** | 0 | 0 | 0 | 0 | 0 | 0 | 0 | 1 | 1 | 1 | 1 | 0 | 0 | 0 | NA | Secondary (renal cancer) |
| **67** | 0 | 0 | 0 | 0 | 0 | 0 | 0 | 0 | 0 | 0 | 0 | 0 | 0 | 0 | NA | Excluded: No IF, but deposits on EM. |
| **68** | 3 | 3 | 3 | 1 | 3 | 3 | 3 | 1 | 1 | 1 | 1 | 2 | 1 | 0 | Pos. | Primary |
| **69** | 1 | 0 | 1 | 0 | 0 | 1 | 1 | 1 | 1 | 1 | 1 | 0 | 0 | 0 | NA | Secondary (endometrial cancer) |
| **70** | 0 | 0 | 0 | 0 | 0 | 0 | 0 | No IHC score | No IHC score | No IHC score | No IHC score | No IHC score | No IHC score | No IHC score | NA | Secondary (Sjögren syndrome) |

*Individual patient-level glomerular PLA₂R immunohistochemistry (IHC) scores (0-3) for all seven observers at AUH and HEH, shown together with serum anti-PLA₂R antibody status and final clinical diagnosis. Each row represents one patient (Patient ID). “No tissue” indicates that no biopsy material was available for re-staining; “No IHC score” indicates that tissue was available, but no valid IHC score could be assigned (e.g. not sufficient glomeruli for scoring). The anti-PLA2R column summarizes serum PLA₂R status (Pos., Neg., NA), and the MN type column classifies patients as primary MN, secondary MN (with underlying condition in parentheses), controls (including day-zero and non-MN diagnoses). Patient ID 31 and 67 were excluded (e.g. discrepant EM findings). Cells are color-coded as follows: green = IHC score ≥2, salmon = IHC score 0-1, grey = ‘No tissue’, yellow = ‘No IHC score’.*
